# Indirect Calorimetry in Spontaneously Breathing, Mechanically Ventilated and Extracorporeally Oxygenated Patients: An Engineering Review

**DOI:** 10.3390/s23084143

**Published:** 2023-04-20

**Authors:** Sebastiaan Priem, Joop Jonckheer, Elisabeth De Waele, Johan Stiens

**Affiliations:** 1 Department of Electronics and Informatics (ETRO), Vrije Universiteit Brussel, Pleinlaan, 1050 Brussels, Belgium; 2 Department of Intensive Care, Universitair Ziekenhuis Brussel, Vrije Universiteit Brussel, Laarbeeklaan, 1090 Brussels, Belgium; 3 Department of Nutrition, Universitair Ziekenhuis Brussel, Vrije Universiteit Brussel, Laarbeeklaan, 1090 Brussels, Belgium

**Keywords:** indirect calorimetry (IC), extracorporeal membrane oxygenation (ECMO), resting energy expenditure (REE), metabolism, oxygen sensor, carbon dioxide sensor, flow sensor, respiratory quotient (RQ)

## Abstract

Indirect calorimetry (IC) is considered the gold standard for measuring resting energy expenditure (REE). This review presents an overview of the different techniques to assess REE with special regard to the use of IC in critically ill patients on extracorporeal membrane oxygenation (ECMO), as well as to the sensors used in commercially available indirect calorimeters. The theoretical and technical aspects of IC in spontaneously breathing subjects and critically ill patients on mechanical ventilation and/or ECMO are covered and a critical review and comparison of the different techniques and sensors is provided. This review also aims to accurately present the physical quantities and mathematical concepts regarding IC to reduce errors and promote consistency in further research. By studying IC on ECMO from an engineering point of view rather than a medical point of view, new problem definitions come into play to further advance these techniques.

## 1. Introduction

Nutritional medical guidelines advocate a personalized approach based on the knowledge of physiological characteristics [1]. In addition to decisions on timing of initiation and on route of delivery (enterally and/or parenterally), the dosing aspect is of cardinal importance. Adequate delivery of energy and proteins will lower mortality in critically ill patients [2,3,4,5]. Resting energy expenditure (REE) needs to be measured to enable a personalized approach. Indirect calorimetry (IC) is currently considered the gold standard as a bedside technique for measuring REE and is recommended by important nutrition and intensive care societies, including the European Society for Clinical Nutrition and Metabolism (ESPEN), the American Society for Parenteral and Enteral Nutrition (ASPEN), and the Society of Critical Care Medicine (SCCM) [1,6,7]. The use of this technique is on the rise and technology has evolved, but many challenges still prohibit use in critically ill patients. A rising clinical hurdle is the concomitant use of treatment modalities in a single patient which induces the exchange of oxygen ($O_{2}$) and/or carbon dioxide (${\mathrm{CO}}_{2}$). In those cases, IC currently fails to measure the $O_{2}$ consumption and/or ${\mathrm{CO}}_{2}$ production of the patient and, therefore, an incorrect REE will be calculated [8].

Extracorporeal membrane oxygenation (ECMO) is such a technique. It consists of a high flow extracorporeal blood circuit driven by a pump and connected to a filter that will oxygenate the blood and remove ${\mathrm{CO}}_{2}$ by means of a sweep gas containing a high amount of $O_{2}$. ECMO is an advanced treatment option for severely ill patients with life threatening pulmonary and/or cardiac failure [9]. The recent COVID-19 pandemic illustrated possible reduction in mortality when used correctly. Nevertheless, as critically ill ECMO patients have survival rates around 50%, treatment challenges remain to improve patient outcome [10]. The use of IC decreases mortality in the general critically ill population, thus should be made feasible during ECMO treatment [2,3]. A first step is a correct understanding of the challenges and pitfalls so that possible remedies can be defined.

The first aim of this review is to provide the reader with a complete theoretical foundation for determining REE by means of IC. Hereby, providing the mathematical concepts and correctly presenting the physical quantities to promote consistency in further research. Secondly, an overview is given of the currently used/proposed techniques and their technical aspects, to perform IC in spontaneously breathing, mechanically ventilated, and extracorporeally oxygenated patients. Special attention is given to the various sensors and their principles used in these IC systems. Finally, the information obtained from these objectives is combined to establish the capabilities and limitations of these systems in an engineering context.

## 2. Theoretical Aspects of Indirect Calorimetry

In this section, the theoretical aspects of IC will be described, using the correct symbols and formulations of the equations, in order to provide the reader with a basic and accurate foundation of IC.

### 2.1. The Weir Formula

REE can be calculated by utilizing the Weir formula. Two different variations of the formula exist. The first variation is based on the principle that 5.941 L of $O_{2}$ has to be metabolized to produce 1 g of urinary nitrogen (*UN*) and is shown in Equation (1) [11,12].

(1) $$REE=(3.941\Delta {\dot{V}}_{O_{2}}+1.106\Delta {\dot{V}}_{CO_{2}}-2.168\Delta {\dot{m}}_{UN})\;\cdot \;1440$$

In this formula, $\Delta {\dot{V}}_{O_{2}}$ and $\Delta {\dot{V}}_{{\mathrm{CO}}_{2}}$ represent $O_{2}$ consumption and ${\mathrm{CO}}_{2}$ production rates, respectively, expressed in L/min and $\Delta {\dot{m}}_{\mathrm{UN}}$ represents the mass of UN in g/min which is a correction factor for protein metabolism [12]. A detailed derivation of the formula can be found in Appendix A.

A second variation of the Weir formula is shown in Equation (2) [12].

(2) $$REE=\frac{3.941\Delta {\dot{V}}_{O_{2}}+1.106\Delta {\dot{V}}_{CO_{2}}}{1-0.0818p}\;\cdot \;1440$$

In this formula, p represents the fraction of caloric production due to protein metabolism. In humans, this fraction is about 12.5%, which means that protein metabolism only results in a deduction of 1.02% of the total REE [12]. Because the contribution of protein metabolism is so little and the relevant parameters are impractical to obtain, this factor is often neglected in the calculations. Therefore, the abbreviated Weir formula is frequently used in clinical practice as shown in Equation (3).

(3) $$REE=(3.941\Delta {\dot{V}}_{O_{2}}+1.106\Delta {\dot{V}}_{CO_{2}})\;\cdot \;1440$$

To determine $\Delta {\dot{V}}_{O_{2}}$ and $\Delta {\dot{V}}_{{\mathrm{CO}}_{2}}$, Equations (4) and (5) are used [13]:

(4) $$\Delta {\dot{V}}_{O_{2}}=\left({\dot{V}}_{i}\;\cdot \;F_{i,O_{2}}\right)-\left({\dot{V}}_{e}\;\cdot \;F_{e,O_{2}}\right)$$

(5) $$\Delta {\dot{V}}_{CO_{2}}=\left({\dot{V}}_{e}\;\cdot \;F_{e,CO_{2}}\right)-\left({\dot{V}}_{i}\;\cdot \;F_{i,CO_{2}}\right)$$

In these equations, ${\dot{V}}_{i}$ and ${\dot{V}}_{e}$ are inhaled and exhaled volumetric flow rates in L/min and $F_{i}$ and $F_{e}$ are volume fractions in inhalation and exhalation, respectively.

### 2.2. The Haldane Transformation

In most modern indirect calorimeters, the inhaled volumetric rate is not measured, but the Haldane transformation is used instead [14]. This transformation relies on the assumption that nitrogen ($N_{2}$) is inert, meaning it is neither produced nor consumed and does not take part in the gas exchange [15]. The use of the Haldane transformation results in Equations (6) and (7) and eliminates the need to measure volumetric flow rates of inhalation. A detailed derivation of these equations can be found in Appendix B.

(6) $$\Delta {\dot{V}}_{O_{2}}={\dot{V}}_{e}\left[\left(\frac{1-F_{e,O_{2}}-F_{e,CO_{2}}}{1-F_{i,O_{2}}-F_{i,CO_{2}}}\right)F_{i,O_{2}}-F_{e,O_{2}}\right]$$

(7) $$\Delta {\dot{V}}_{CO_{2}}={\dot{V}}_{e}\left[F_{e,CO_{2}}-\left(\frac{1-F_{e,O_{2}}-F_{e,CO_{2}}}{1-F_{i,O_{2}}-F_{i,CO_{2}}}\right)F_{i,CO_{2}}\right]$$

The values calculated for $\Delta {\dot{V}}_{O_{2}}$ and $\Delta {\dot{V}}_{{\mathrm{CO}}_{2}}$ can, subsequently, be substituted into Equation (3) to calculate REE. Note that $F_{i,{\mathrm{CO}}_{2}}$ is equal to only 0.03–0.05% and is, by consequence, often neglected [8].

## 3. Technical Aspects of Indirect Calorimetry

Two main techniques to perform IC exist, closed-circuit and open-circuit. In closed-circuit IC, there is no connection between the ambient air and the patient and a distinction can be made between two sub-types, the volume-loss and the volume-replenishment technique. In contrast to the closed-circuit method, open-circuit IC does have a connection between the patient and the ambient air. Three main sub-techniques exist, the mixing chamber, dilution, and breath-by-breath technique. These different methods and their technical aspects are presented in this section.

### 3.1. Closed-Circuit Indirect Calorimetry

**Figure 1:**
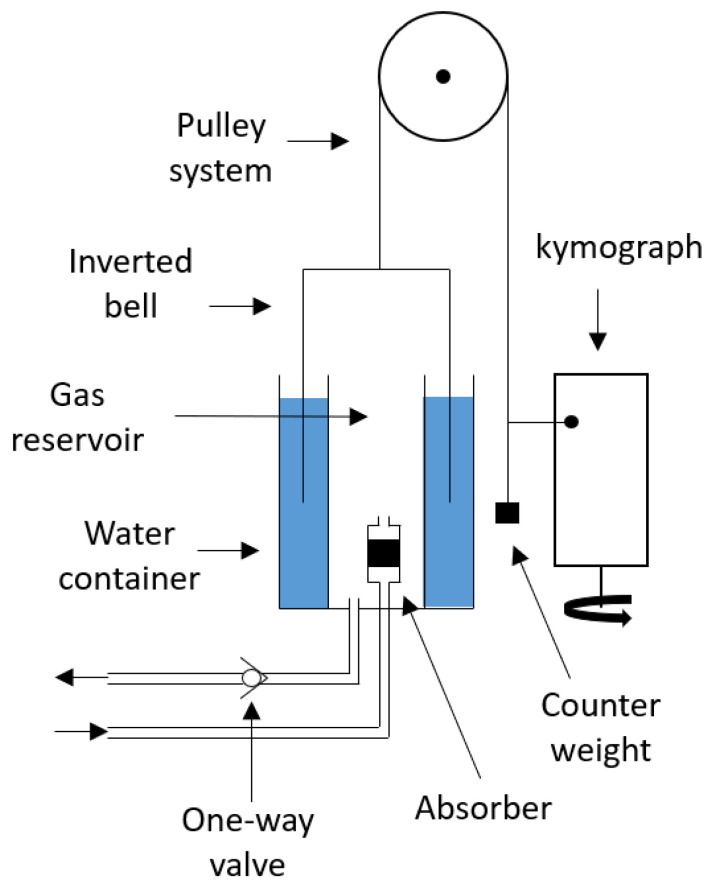
Benedict–Roth spirometer used for the determination of the $O_{2}$ consumption rate of patients.

The volume-loss technique, illustrated in Figure 1, utilizes a spirometer, usually the Benedict–Roth spirometer. The device consists of a cylindrical water container and an inverted, cylindrical bell which is connected to a pulley system with a counterweight to compensate for the effect of buoyancy on the weight of the bell as it moves vertically in the water [16]. Subsequently, a pen, attached to the pulley system, writes onto a kymograph to record the change in volume of the spirometer. When performing the volume loss technique, the patient breaths in gas from a reservoir containing a known amount of $O_{2}$ through the combination of a mouthpiece and a nose clip or a face mask. After inspiration, a portion of the $O_{2}$ is consumed by the patient and ${\mathrm{CO}}_{2}$ is produced. When exhaling, the produced ${\mathrm{CO}}_{2}$ and water vapor is absorbed, usually by potassium hydroxide, and the residual gas is redirected to the reservoir. Subsequently, $\Delta {\dot{V}}_{O_{2}}$ is determined by the volume loss of the spirometer over time [17]. Note that a one-way valve is present in the inspiratory limb to prevent the exhaled air bypassing the ${\mathrm{CO}}_{2}$ absorber.

$\Delta {\dot{V}}_{{\mathrm{CO}}_{2}}$ can be estimated because the respiratory quotient (RQ), being the ratio of carbon dioxide produced and oxygen consumed, is chosen as a constant value and multiplied with the calculated $O_{2}$ consumption rate [16]. For a typical subject on a typical diet RQ is between 0.8 and 0.85 [17].

Another type of closed-circuit technique is the volume-replenishment technique. This technique is similar to the volume-loss technique, but now a constant spirometer volume is maintained as $O_{2}$ is reinserted into the system. The amount of $O_{2}$ that is replenished is equivalent to the consumed volume of $O_{2}$ [18]. Note that in both techniques a thermometer is placed inside the bell. The reason is that a gas contracts or expands in volume as it is cooled or heated, respectively, as described by Charles’ law [19]. By monitoring the temperature inside the bell, this problem can be compensated for.

### 3.2. Open-Circuit Technique

**Figure 2:**
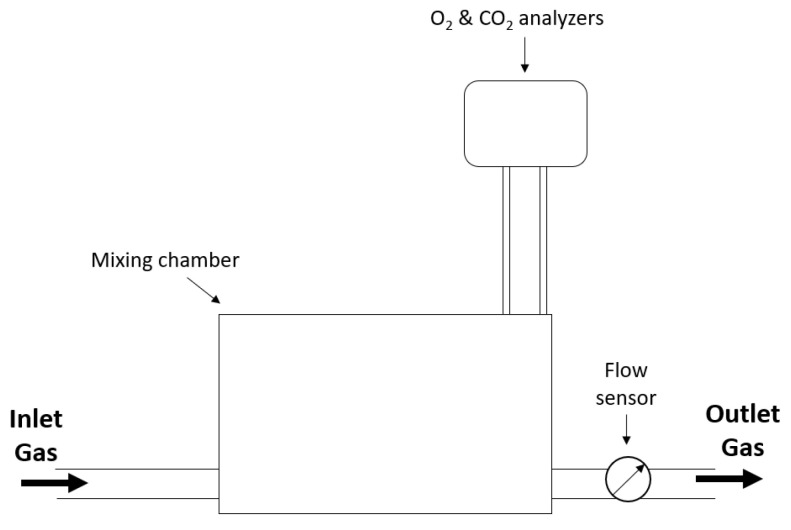
Generic open-circuit indirect calorimeter using the mixing chamber technique with the bold arrows indicating the direction of gas flow.

Most modern indirect calorimeters make use of a mixing chamber; a generic system is shown in Figure 2.

The expired gas from the patient is directed to the mixing chamber. A sampling pump draws a sample of gas out of the mixing chamber and directs it to the gas analyzers to determine $O_{2}$ and ${\mathrm{CO}}_{2}$ concentrations. After gas analysis, the sample is returned to the mixing chamber where it exits the system while the volumetric flow rate is being measured [20]. The inspired gas is analyzed by the same gas analyzers at predetermined intervals or continuously by a second set of analyzers. The purpose of the mixing chamber is to make the indirect calorimeter less susceptible to outliers by adding a new breath to a pool of breaths previously collected, resulting in less variability in the measurements [21].

**Figure 3:**
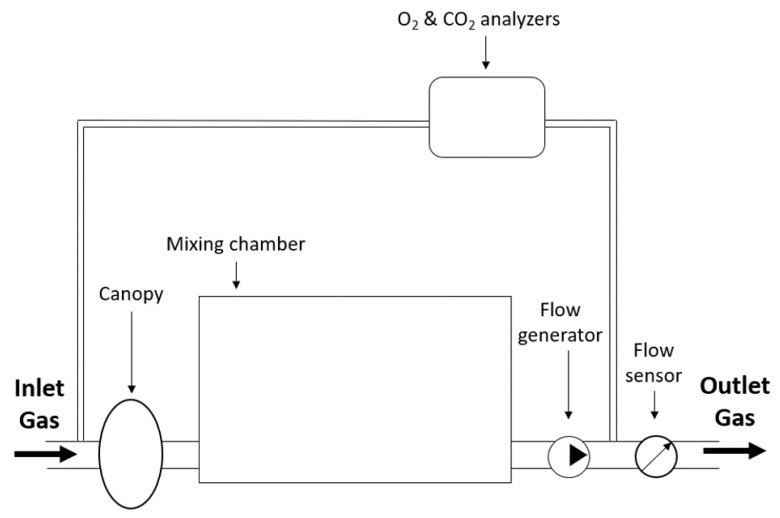
Schematic representation of the open-circuit dilution technique for calculating REE in spontaneously breathing subjects with the bold arrows indicating the direction of gas flow.

An adaption of the open-circuit mixing chamber technique is the open-circuit dilution technique. This technique can be used to assess REE in spontaneously breathing patients, as well as in mechanically ventilated patients, as schematically illustrated in Figure 3.

In spontaneously breathing patients, a canopy hood is placed over the patient’s head, often sealed with a drape to avoid air leaks. Subsequently, the surrounding air is drawn into the hood by means of a flow generator of which the rotation speed can be adjusted [8,13]. The concentrations of $O_{2}$ and ${\mathrm{CO}}_{2}$ in the surrounding air are closely monitored as they represent $F_{i,O_{2}}$ and $F_{i,{\mathrm{CO}}_{2}}$. Subsequently, the patient’s breaths are diluted with the surrounding air and the mixture is drawn into a mixing chamber, by the ventilator, in which the diluted air is physically averaged to provide more stable measurements and to reduce the coefficient of variation in $\Delta {\dot{V}}_{O_{2}}$ and $\Delta {\dot{V}}_{{\mathrm{CO}}_{2}}$ [22]. Next, a sample of air from the mixing chamber is drawn into the gas analyzers and diluted volume fractions of $O_{2}$ and ${\mathrm{CO}}_{2}$, $F_{d,O_{2}}$ and $F_{d,{\mathrm{CO}}_{2}}$, are measured. The calculations of ${\bar{F}}_{e,O_{2}}$ and ${\bar{F}}_{e,{\mathrm{CO}}_{2}}$ are usually completed by means of Equations (8) and (9), respectively, in which *T* is the period of time over which the averaging is completed [23].

(8) $${\bar{F}}_{e,O_{2}}=\frac{1}{T}\int_{0}^{T}\left(F_{i,O_{2}}\left(t\right)-F_{d,O_{2}}\left(t\right)\right)\;dt$$

(9) $${\bar{F}}_{e,CO_{2}}=\frac{1}{T}\int_{0}^{T}\left(F_{d,CO_{2}}\left(t\right)-F_{i,CO_{2}}\left(t\right)\right)\;dt$$

These volume fractions can then be used to calculate $\Delta {\dot{V}}_{O_{2}}$ and $\Delta {\dot{V}}_{{\mathrm{CO}}_{2}}$ of the patient by using Equations (6) and (7). Finally, the Weir equation can be used to calculate REE. The most important advantage of this technique is that ${\dot{V}}_{e}$ can be replaced with the constant flow rate, ${\dot{V}}_{const}$, generated by the constant flow ventilator. It is important that the rotation speed of the ventilator must be high enough to prevent a build-up of ${\mathrm{CO}}_{2}$ inside the canopy, which can cause hypercapnia resulting in discomfort for the patient and a more erroneous result of the measurement. Note that, instead of a canopy, the clinician can also opt to use a face-mask for patients who are not comfortable using a hood [24].

**Figure 4:**
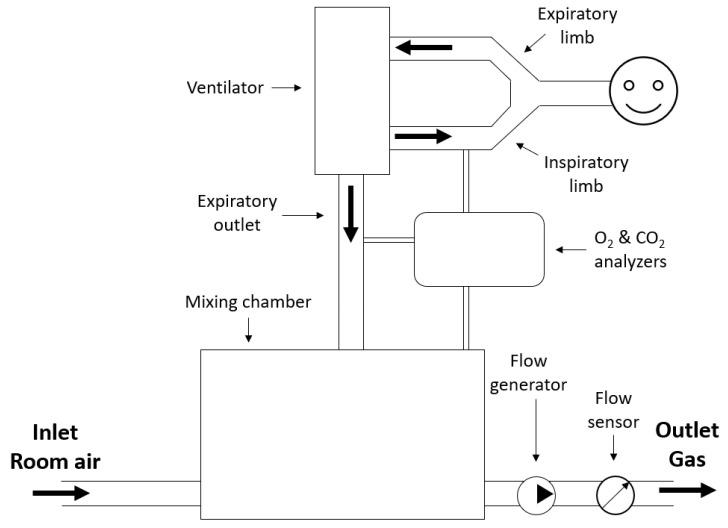
Schematic representation of the open-circuit technique for calculating REE in mechanically ventilated patients with the bold arrows indicating the direction of gas flow.

This technique, with some adaptations, can also be used to measure REE in mechanically ventilated patients [23]. The schematic representation of the open-circuit dilution technique for ventilated patients is shown in Figure 4.

The difference with the application of the technique for spontaneously breathing patients is that now three gas measurements are taken, at the inspiratory limb, the expiratory outlet, and at the mixing chamber. $\Delta {\dot{V}}_{{\mathrm{CO}}_{2}}$ is calculated using Equation (10) [23].

(10) $$\Delta {\dot{V}}_{CO_{2}}={\dot{V}}_{const}\;\cdot \;F_{d,CO_{2}}$$

Subsequently, the RQ is calculated by taking the ratio of $\Delta {\dot{V}}_{{\mathrm{CO}}_{2}}$ and $\Delta {\dot{V}}_{O_{2}}$, neglecting $F_{i,{\mathrm{CO}}_{2}}$, as shown in Equation (11).

(11) $$RQ=\frac{\Delta {\dot{V}}_{CO_{2}}}{\Delta {\dot{V}}_{O_{2}}}=\frac{1-F_{i,O_{2}}}{\frac{F_{i,O_{2}}-F_{e,O_{2}}}{F_{e,CO_{2}}}-F_{i,O_{2}}}$$

Subsequently, from the definition of RQ, $\Delta {\dot{V}}_{O_{2}}$ can be calculated and be substituted in the formula of Weir to calculate REE.

The third open-circuit technique is the breath-by-breath technique. This technique utilizes the same formulas as the generic open-circuit mixing chamber technique, but the major difference is the absence of a mixing chamber. Consequently, the averaging of the gases is performed by software instead of physically, intra-breath profiles are identified and each respiratory cycle is analyzed [20].

## 4. Indirect Calorimetry in Patients Undergoing ECMO

In this section, the current methods for the application of IC in patients undergoing ECMO will be discussed. The two methods currently proposed are based on traditional IC by performing gas analysis and on a combination of traditional IC and blood gas measurements.

### 4.1. Traditional Indirect Calorimetry in Patients Undergoing ECMO

De Waele et al. (2019) proposed a theoretical method to assess the REE in critically ill patients undergoing ECMO based on the principle that there are two sides of gas exchange, the natural lung and the oxygenator compartment of the ECMO-circuit [25]. The proposed technique consists of performing traditional IC measurements on both the natural lung and the membrane lung of the ECMO-circuit sequentially. As patients on ECMO are usually mechanically ventilated, the IC measurement at the natural lung is performed as in any other patient on mechanical ventilation [26]. Subsequently, the measurement at the membrane lung is taken by directing samples of sweep gas from the gas inlet and the gas outlet of the oxygenator to the same indirect calorimeter after the measurement at the natural lung is performed. By adding the $O_{2}$ consumption rate measured at the natural lung ($\Delta {\dot{V}}_{O_{2},lung}$) and the $O_{2}$ consumption rate measured at the oxygenator ($\Delta {\dot{V}}_{O_{2},ECMO}$), a total $O_{2}$ consumption rate ($\Delta {\dot{V}}_{O_{2},tot}$) can be determined as shown in Equation (12).

(12) $$\Delta {\dot{V}}_{O_{2},tot}=\Delta {\dot{V}}_{O_{2},lung}+\Delta {\dot{V}}_{O_{2},ECMO}$$

Subsequently, a total ${\mathrm{CO}}_{2}$ production rate ($\Delta {\dot{V}}_{{\mathrm{CO}}_{2},tot}$) is calculated in a similar manner as shown in Equation (13).

(13) $$\Delta {\dot{V}}_{CO_{2},tot}=\Delta {\dot{V}}_{CO_{2},lung}+\Delta {\dot{V}}_{CO_{2},ECMO}$$

Finally, these values are inserted into the formula of Weir to calculate the total REE of the patient.

### 4.2. A Combination of Traditional Indirect Calorimetry and Blood Gas Analysis in Patients Undergoing ECMO

T. Wollersheim et al. (2018) developed a method for measuring REE based on the same principle as De Waele et al. (2019), namely that there are two sides of gas exchange in ECMO patients, referred to as “Measuring Energy Expenditure in extracorporeal lung support Patients” (MEEP) [25,27]. The measurement of gas exchange at the natural lung side is still performed by using traditional IC, but gas exchange parameters at the membrane lung are determined by taking blood samples before and after the oxygenator. Subsequently, blood gas analysis (BGA) is performed on these samples. Next, the results of the BGA are inserted into the model of Dash and Bassingthwaighte, which is a mathematical model that calculates $O_{2}$ and ${\mathrm{CO}}_{2}$ concentrations in the blood [28]. $\Delta {\dot{V}}_{O_{2}}$ and $\Delta {\dot{V}}_{{\mathrm{CO}}_{2}}$ can be determined by multiplying the difference of $O_{2}$ ($\Delta c_{O_{2}}$) and ${\mathrm{CO}}_{2}$ concentrations ($\Delta c_{{\mathrm{CO}}_{2}}$), in volume percentages, with the volumetric blood flow in the ECMO-circuit (${\dot{V}}_{blood}$), as shown in Equations (14)–(17).

(14) $$\Delta c_{O_{2}}=c_{O_{2},post}-c_{O_{2},pre}$$

(15) $$\Delta c_{CO_{2}}=c_{CO_{2},pre}-c_{CO_{2},post}$$

(16) $$\Delta {\dot{V}}_{O_{2},ECMO}=\Delta c_{O_{2}}\;\cdot \;{\dot{V}}_{blood}$$

(17) $$\Delta {\dot{V}}_{CO_{2},ECMO}=\Delta c_{CO_{2}}\;\cdot \;{\dot{V}}_{blood}$$

After this, $\Delta {\dot{V}}_{O_{2},tot}$ and $\Delta {\dot{V}}_{{\mathrm{CO}}_{2},tot}$ are again calculated by summing the determined consumption/production rates of both sides as performed in Equations (12) and (13). Finally, by substituting the calculated values for $\Delta {\dot{V}}_{O_{2},tot}$ and $\Delta {\dot{V}}_{{\mathrm{CO}}_{2},tot}$ in the formula of Weir, as shown in Equation (3), the REE can be calculated.

## 5. Sensors Used in Indirect Calorimeters

Commercially available indirect calorimeters use different sensors to measure the various parameters required to determine REE (e.g., $O_{2}$ and ${\mathrm{CO}}_{2}$ concentrations and volumetric flow rate). Note that different subgroups of patients often result in the necessity to measure a different range of values for the above depicted parameters. In this section, the measurement principles of these sensors are described.

### 5.1. Oxygen Analyzer

Typical gas analyzers used for measuring $O_{2}$ concentrations in IC are the paramagnetic oxygen sensor and the galvanic fuel cell (GFC) [29].

**Figure 5:**
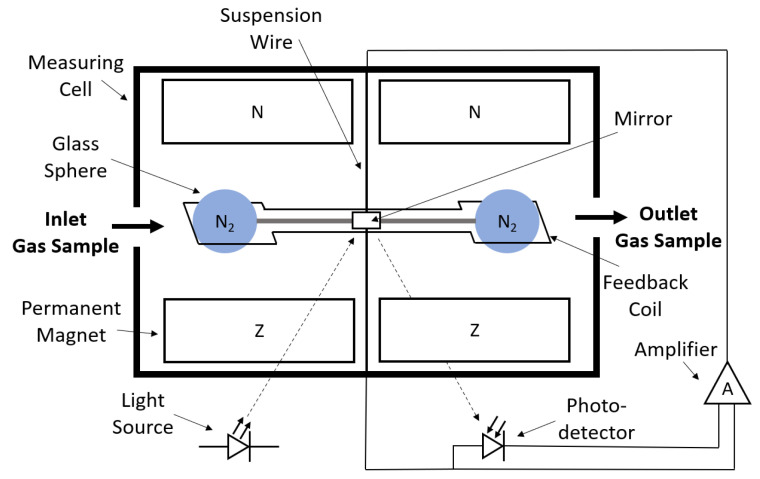
Schematic drawing of a paramagnetic oxygen sensor consisting of two glass spheres filled with $N_{2}$, a permanent magnet, a mirror, a suspension wire, a feedback coil, a light source, and a photodetector with the bold arrows indicating the direction of gas flow and the dotted arrows indicating the direction of light.

$O_{2}$ is paramagnetic which means that it is weakly attracted to an externally applied magnetic field. This property is the basis of the working principle of the paramagnetic oxygen sensor. A first type of paramagnetic oxygen sensor consists of two glass spheres filled with $N_{2}$, which is a diamagnetic substance, a suspension wire, a feedback coil, a mirror that is mechanically attached to these spheres, and a light source and a detector or photo-sensor. The described setup is exposed to a magnetic field, as shown in Figure 5.

In the initial state, the light source shines on the mirror and the beam is reflected onto the detector. This signal is converted to an electrical current which is redirected to the feedback coil. Subsequently, the feedback coil produces a magnetic field which will interact with the initial magnetic field from the permanent magnet causing the glass spheres to remain in a stable position. When $O_{2}$ enters the measurement cell, $O_{2}$ molecules will be attracted to the existing magnetic field, resulting in a force acting on the glass spheres, eventually causing the spheres to rotate. Because the mirror is mechanically attached to the spheres it will also rotate leading to a change in the amount of light that is reflected back to the detector. Subsequently, the current in the feedback coil will try to create a counter torque to return the spheres back in their original position. The change in feedback current is directly proportional, after proper calibration, to the concentration of $O_{2}$ entering the measurement cell [30,31,32]. Typical response time of this type of oxygen sensor is 1–10 s, accuracy and resolution values are reported of 100 ppm in the range of 10–100% oxygen [31,33].

**Figure 6:**
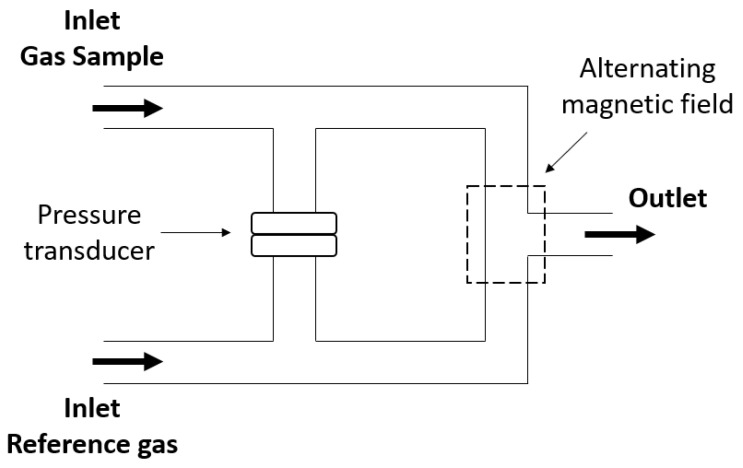
Schematic drawing of a paramagnetic oxygen sensor consisting of two gas inlets, sample and reference, a pressure transducer and an electromagnet to generate an alternating magnetic field with the bold arrows indicating the direction of gas flow.

More modern paramagnetic oxygen analyzers consist of two separate inlets, one for the breathing gas and one for the reference gas (e.g., room air), a pressure transducer, and an electromagnet, as illustrated in Figure 6.

The electromagnet is used to create a rapidly switching magnetic field, 100–110 Hz, which attracts the $O_{2}$ molecules and causes them to be agitated [34]. Consequently, the pressure on each side that contains $O_{2}$ will change and this pressure difference, measured by the pressure transducer, is proportional to the difference in $O_{2}$ partial pressure between the breathing and the reference gas [35]. According to scholarly sources, it has been reported that paramagnetic oxygen sensors exhibit a faster response time compared to dumbbell-type oxygen sensors, in the range of hundreds of ms [36,37,38] and an accuracy of <1% has been achieved [31,37].

**Figure 7:**
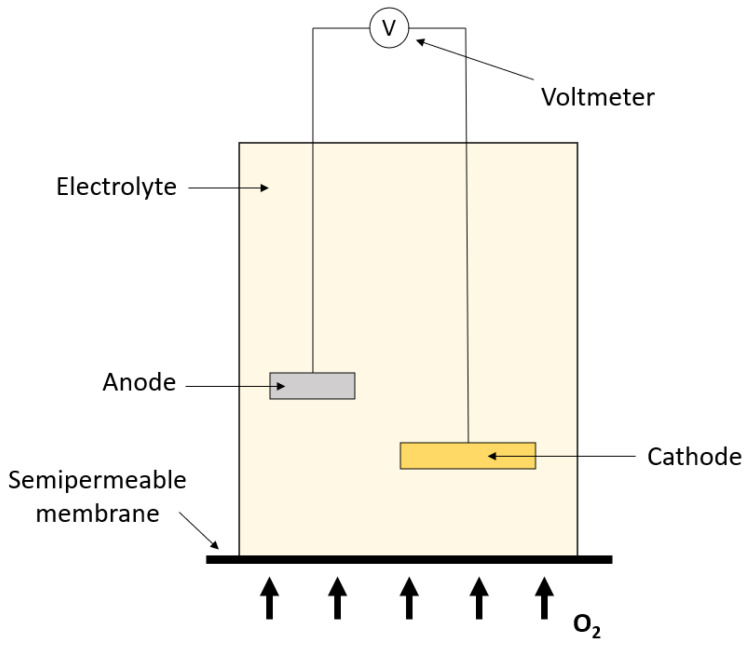
Schematic drawing of a galvanic fuel cell (GFC) oxygen analyzer.

Another type of oxygen sensor used in IC is the GFC. This type of oxygen sensor consists of a cathode made of a precious metal (e.g., gold, silver, platinum, etc.), an electrolyte (e.g., potassium hydroxide), an anode made of a base material (e.g., lead, cadmium, etc.) and a permeable membrane to $O_{2}$, but not to the electrolyte (e.g., Teflon or silicone) [39]. A schematic diagram of the GFC is illustrated in Figure 7.

When $O_{2}$ molecules enter the measurement cell, by diffusion through the membrane, they are reduced at the cathode to hydroxide ions and are subsequently oxidized at the anode. The cathode, anode and overall reaction in case of a lead anode are shown in Reaction (R 1) to (R 3), respectively.

(R 1) $$O_{2}+2H_{2}O+4e^{-}\to 4OH^{-}$$

(R 2) $$2Pb+4OH^{-}\to 2PbO+2H_{2}O+4e^{-}$$

(R 3) $$2Pb+O_{2}\to 2PbO$$

These reactions will eventually generate an electric potential which is proportional to the $O_{2}$ concentration in the sample gas [40]. According to the literature, the response time (T90) of these sensors can be as fast as 2.7 s in the range of 21–100% $O_{2}$ [41,42,43] and accuracies are reported as 1–3% of the reading [44,45]. High-resolution values are reported by manufacturers as fine as 0.0001% $O_{2}$, e.g., in the FC-10 Oxygen Analyzer of Sable Systems International [46].

An overview of oxygen sensors used in commercial indirect calorimeters with their corresponding technical specifications as provided by the manufacturers is given in Table 1.

Note that, in most cases, the reported specifications of the manufacturer are better than the stated values reported in literature [41,42,43,44,45]. In particular, the provided specifications of T90 response time and accuracy of the GFC type $O_{2}$ sensors in commercial indirect calorimeters are remarkably better then what literature reports. This suggests that independent validity studies of these sensors are advisable.

### 5.2. Carbon Dioxide Analyzer

**Figure 8:**
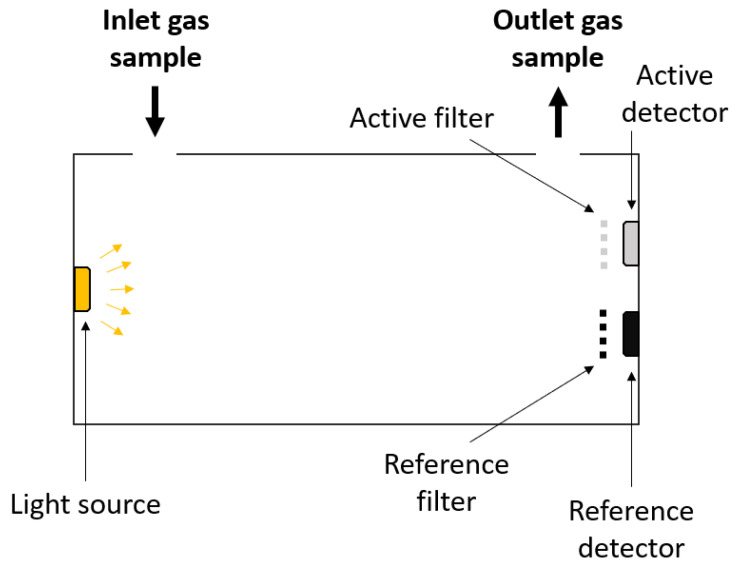
Schematic drawing of a non-dispersive infrared (NDIR) carbon dioxide sensor.

To measure levels of ${\mathrm{CO}}_{2}$, most modern indirect calorimeters use a non-dispersive infrared (NDIR) carbon dioxide sensor [53,54]. These sensors consist of a light source, two optical filters (active and reference) and two photodetectors, as shown in Figure 8. These sensors rely on the measurement of light intensities [55].

A relationship exists between the concentration of a gas and the absorbance of light as it propagates through that gas. This relationship is given by the Beer–Lambert law shown in Equation (18) [56].

(18) $$T=\frac{I}{I_{0}}=e^{-\epsilon cL}$$

*T* represents the transmittance, $I_{0}$ and *I* represent the light intensity at the source and at the detector, respectively, ϵ is the molar absorption coefficient of the gas at a given wavelength, c is the concentration of the gas, and L represents the optical path length. When ${\mathrm{CO}}_{2}$ enters the measuring cell, it will absorb some of the light from the light source. ${\mathrm{CO}}_{2}$ has a strong absorption band about 4.3 μm [57,58]. Subsequently, the attenuated light will pass through the active filter, which allows light with a wavelength of about 4.3 μm, and, eventually, it will reach the detector. By using Equation (18), the concentration of the gas, e.g., ${\mathrm{CO}}_{2}$, can be determined. The reference filter allows light with a wavelength of about 3.9 μm. In this range, no common molecules absorb [58]. The function of the reference channel is to eliminate measuring errors caused by dust or degradation of the light source. The response time of these sensors is dependent on the flow rate of the gas passing through the measuring cell. NDIR ${\mathrm{CO}}_{2}$ sensors exist, e.g., SprintIR-6S, with a response time (T90) of 300 ms at a flow rate of 1 L/min, an accuracy of ±70 ppm + 5% of reading and a resolution of 10 ppm ${\mathrm{CO}}_{2}$ as reported by the manufacturer (GSS Sensing Solutions) [59].

Table 2 presents a comprehensive summary of the technical specifications of NDIR ${\mathrm{CO}}_{2}$ sensors used in commercial indirect calorimeters as reported by their respective manufacturers.

### 5.3. Flow Meter

Two types of flow meters are used in commercially available indirect calorimeters, the turbine flow meter and the Pneumotachometer.

**Figure 9:**
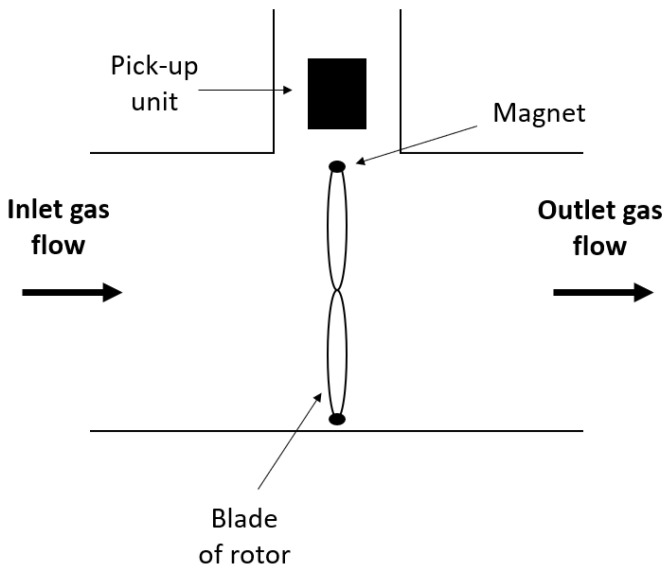
Schematic drawing of an inductive turbine flow sensor.

Turbine flow meters generally consist of a mechanical (rotor) and an electrical part (pick-up unit). Two variants, inductive and optical, exist [60]. An inductive turbine flow meter is illustrated in Figure 9.

When gas enters the measurement chamber it will strike the blades of the rotor, causing it to start rotating. Consequently, the rotational speed of the rotor is proportional to the volumetric flow rate of the gas [61]. Small magnets, usually incorporated in the blades, induce a magnetic field in the surrounding area. The pick-up unit, e.g., a Hall sensor or a pick-up coil with permanent magnet, will sense this magnetic field and produce an electrical signal in the form of pulses. The number of pulses per unit of time will be proportional to the varying magnetic field and, consequently, to the volumetric flow rate of the gas [62].

More modern types of turbine flow meters may use optical sensors to detect rotations of the rotor. In other words, the measurement principle is the same as in the inductive variant, but magnets are unnecessary and the pick-up unit is now an optical fiber probe [63].

Some commercially available indirect calorimeters that use a turbine flow sensor are the Q-NRG, Q-NRG+, and the Quark RMR (Cosmed). Typical accuracy is ±1–2% [64].

**Figure 10:**
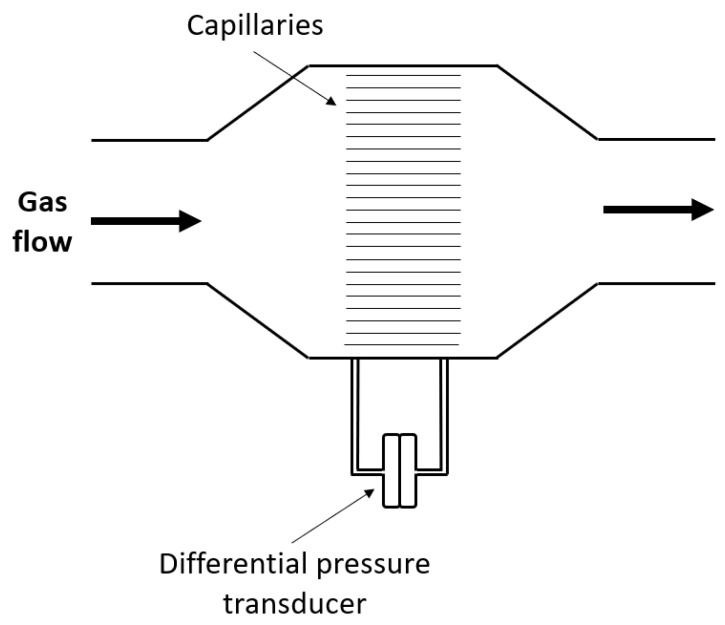
Schematic drawing of a Fleisch Pneumotachometer.

The second type of flow meter that is regularly used in indirect calorimeters is the Pneumotachometer. Mainly two variations exist, the Fleisch Pneumotachometer and the Lilly Pneumotachometer. The Fleisch type is composed of capillary tubes and a differential pressure transducer, as illustrated in Figure 10.

The measurement principle relies on the Hagen–Poiseuille law shown in Equation (19) [65].

(19) $$\dot{V}=\frac{\pi D^{4}\Delta P}{128\mu L}$$

$\dot{V}$ is the volumetric flow rate (note that Q is also commonly used), *D* is the diameter of the tube, ΔP is the pressure difference between the two sides of the tube, µ is the dynamic viscosity of the measured substance and L is the length of the tube. When the gas flows through the flow meter it enters an array of capillaries. These capillaries create a small resistance resulting in a pressure drop between both sides of the sensor. The pressure drop over the capillaries is measured and is directly proportional to the volumetric flow rate of the gas as shown by Equation (19). The secondary function of these capillaries is to keep the flow laminar [66]. The Lilly Pneumotachometer uses the same principle, but utilizes a fine wire mesh [67]. Usually a heating element is incorporated in the Pneumotachometer to prevent the accumulation of moisture due to the condensation of water vapor [68]. This is necessary because moisture can increase the resistivity of the resistive element (mesh or capillaries). Commercially available indirect calorimeters that use these types of flow sensors are the Q-NRG+ (Cosmed), the Quark RMR (Cosmed), and the TrueOne2400 (Parvomedics). Note that the Q-NRG+ and the Quark RMR use both a turbine flow meter and a Pneumotachometer. The performance (response time, resolution, accuracy) of such a Pneumotachometer is highly dependent on the characteristics of the pressure transducer [69]. A response time and resolution of, respectively, <1 m/s and 0.4 L/min have been reported and the accuracy is generally ±2% [70,71].

The technical specifications of flow meters utilized in commercial indirect calorimeters are summarized in Table 3, as reported by the manufacturers. The response time (T90) and resolution are not included as none of the manufacturers reported this information.

## 6. Discussion

In this section the different techniques for performing IC will be discussed and compared, including the proposed techniques for patients undergoing ECMO. In a second part, the limitations and advantages of the different sensors used in IC are described, as well as comparisons between the sensors are provided.

### 6.1. Comparison between the Open- and Closed-Circuit Indirect Calorimetry Techniques

**Figure 11:**
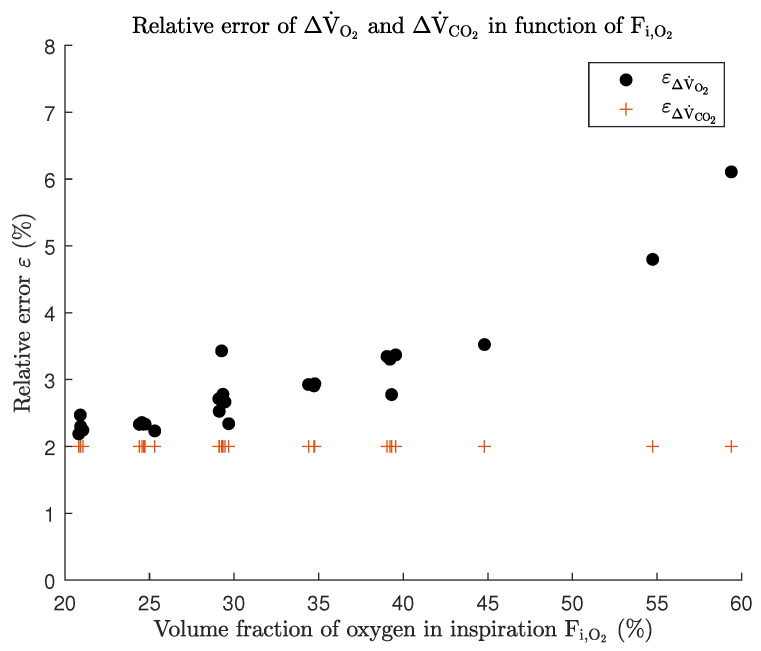
The relative error ε in % of $\Delta {\dot{V}}_{O_{2}}$ (dots) and $\Delta {\dot{V}}_{{\mathrm{CO}}_{2}}$ (plus signs) in function of $F_{i,O_{2}}$ in volume % when using the Haldane transformation.

The primary distinction between the two IC approaches is that in closed-circuit IC there is no direct contact between the external air and the calorimeter, while this is always the case for open-circuit IC. Another significant differentiation between the two techniques concerns the components that are utilized. The open-circuit technique uses gas analyzers and flow meters, while the closed-circuit technique relies on the volume displacement of the gas reservoir recorded by the kymograph. The first and major limitation of traditional open-circuit IC is the error imposed at high levels of $F_{i,O_{2}}$, which are often used in ECMO patients or mechanically ventilated patients in general. Literature suggests that this is due to the usage of the Haldane transformation. To quantify this issue, an error analysis, according to the rules of propagation of uncertainty with data collected from mechanically ventilated patients using the commercial indirect calorimeter Q-NRG+ (Cosmed) is conducted and shown in Figure 11. The data consisted of 25 datasets. Figure 11 shows data of the first accepted measurement by the indirect calorimeter of each of the 25 datasets. Note that a maximum $F_{i,O_{2}}$ of about 60% is used, no higher values have been analyzed due to a lack of data.

The error on $\Delta {\dot{V}}_{{\mathrm{CO}}_{2}}$ remains stable at about 2%, but the error on $\Delta {\dot{V}}_{O_{2}}$ shows an increasing trend, up to about 6.1%, when higher levels of $F_{i,O_{2}}$ are used. Similar conclusions are drawn in [72,73]. Note that the Haldane transformation cannot be used with an $F_{i,O_{2}}$ of 100% because, in this case, no $N_{2}$ is present in the gas. Consequently, Equation (A10) cannot be utilized. In clinical practice, it is recommended to only conduct measurements in mechanically ventilated patients with a maximum $F_{i,O_{2}}$ of about 70%. In vitro validation studies of modern indirect calorimeters using the open-circuit technique, such as the Q-NRG+ from Cosmed, show that the values obtained for $\Delta {\dot{V}}_{O_{2}}$ and $\Delta {\dot{V}}_{{\mathrm{CO}}_{2}}$ were within 5% of the reference values that were obtained with mass spectrometry when simulating gas exchange rates of 150, 250, and 400 mL/min STPD at $F_{i,O_{2}}$ levels of 21, 40, 60, and 70% [74]. Another drawback is that long term continuous measurement are currently impossible because of water vapor condensation of the saturated expired air resulting in a negative effect on the sensor performance (e.g., the Pneumotachometer) and causing an accumulation of water in the tubing of the indirect calorimeter [50,51,75,76]. It is shown that there could be statistically significant intraday variability regarding REE in critically ill patients [77,78]; therefore, long term continuous measurements could be interesting, although clinical significance needs to be explored.

Despite the fact that the closed-circuit technique is not used any longer in modern calorimeters, the problem of an $F_{i,O_{2}}$ of 100% does not occur when utilizing this technique as the absence of gas analyzers eliminate the need to use the Haldane transformation, which is the major advantage of this technique [18,79]. A first limitation of the closed-circuit volume-loss technique is that the amount of $O_{2}$ in the spirometer will decrease over time leading to the delivery of hypoxic gas to the patient [18]. The volume-replenishment technique eliminates this issue as $O_{2}$ is constantly redelivered to the reservoir. Another limitation is that measurements can only last a certain amount of time before the volume of the system has to be refilled. Again, the volume-replenishment technique offers a solution, but the system is still limited by the capacity of the ${\mathrm{CO}}_{2}$ absorber [18]. An additional drawback in closed-circuit IC is that the system is affected by changes in lung volume as a decrease or increase will reflect in an apparent decrease or increase, respectively, in $\Delta {\dot{V}}_{O_{2}}$ [14,18]. Leaks will also affect the measurements because any leak will be interpreted as a change in $\Delta {\dot{V}}_{O_{2}}$ by the system which can lead to significant errors [14]. In addition, the breathing resistance is increased, causing an increase in work of breathing which can result in an elevated REE of the patient. Note that the dead space, the volume of ventilated air that does not participate in the gas exchange, should be minimized or be compensated for by means of a correction factor as it will also influence the measurements [18]. Finally, the utilization of a fixed RQ to approximate $\Delta {\dot{V}}_{{\mathrm{CO}}_{2}}$ can have a detrimental effect on the precision of the IC measurements, and also renders it impossible to determine the REE in individuals who exhibit non-standard RQ values.

### 6.2. Comparison between Mixing Chamber and Breath-by-Breath

In open-circuit IC, the methods using a mixing chamber are considered the best established methods [80], mainly because of the methodological implications in breath-by-breath measurements.

The first implication is the delay time between the measurement of the volume fractions of $O_{2}$ and ${\mathrm{CO}}_{2}$ and the flow signal [81]. This delay time consists of the transport time of the gas to the different sensors and the response time of the sensors. For example, with E-sCOVX (GE Healthcare), the flow signal is measured almost instantaneously (<10 ms), but it takes about 1.5 s for the gas to reach the gas analyzers, also this transport time is not a constant parameter [82]. It highly depends on the configuration of the clinical setup (gas sampling tubing, settings of the flow generator of the indirect calorimeter, mechanical ventilator settings, etc.). The misalignment of these signals results in significant errors on $\Delta {\dot{V}}_{O_{2}}$ and $\Delta {\dot{V}}_{{\mathrm{CO}}_{2}}$ as reported by various studies [83,84]. For example, a study by Bernard reported that a time delay of 200 ms between first measuring $F_{e,CO_{2}}$ and then ${\dot{V}}_{e}$ could have an underestimation of more than 40% on the calculation of $\Delta {\dot{V}}_{{\mathrm{CO}}_{2}}$ [85]. Consequently, there is need for highly precise temporal alignment between the different signals. Additionally, the gas analyzers should possess fast enough response times to take enough sample points in the intra-breath profile to produce a viable result for $\Delta {\dot{V}}_{O_{2}}$ and $\Delta {\dot{V}}_{{\mathrm{CO}}_{2}}$. Studies showed that systems with a sampling frequency lower than 15 Hz and a response time (T90) higher than 100 ms should be avoided [86]. Note that the system should also be able to distinguish between a breath and other phenomena (e.g., a cough).

The techniques utilizing a mixing chamber eliminate these issues because they do not use the integration of the product of continuously measured flow rates and gas concentrations, but they physically average these concentrations before making the necessary calculations to determine $\Delta {\dot{V}}_{O_{2}}$ and $\Delta {\dot{V}}_{{\mathrm{CO}}_{2}}$.

In conclusion, the mixing chamber techniques show higher accuracy and the breath-by-breath techniques show higher temporal resolution [87].

### 6.3. Comparison of Current Indirect Calorimetry Techniques for Patients Undergoing ECMO

The main advantage of using MEEP over the traditional IC technique in patients on ECMO is the ability to measure $\Delta {\dot{V}}_{O_{2},ECMO}$ and $\Delta {\dot{V}}_{{\mathrm{CO}}_{2},ECMO}$ when $F_{i,O_{2}}$ in the sweep gas is higher than 70%. This is almost always the case as the sweep gas usually consists of 100% $O_{2}$ or a mix of $O_{2}$ and ${\mathrm{CO}}_{2}$ (0–5%) [88].

It could be argued that MEEP is less or more labor intensive for the staff. On the one hand, the need to perform two traditional IC measurements is eliminated, but, on the other hand, the blood gases need to be taken and analyzed in addition to performing one traditional measurement.

Another limitation of the traditional IC approach is that there is a delay between the measurement at the natural lung and the measurement at the membrane lung, which can cause erroneous results when calculating $\Delta {\dot{V}}_{O_{2},tot}$ and $\Delta {\dot{V}}_{CO_{2},tot}$. By consequence, it would be better if two identical indirect calorimeters were used at exactly the same time and the measurements lasted exactly the same duration.

The first group of limitations of MEEP is related to time synchronization issues. When performing MEEP, in contrast to traditional IC, only one point measurement is taken before and after oxygenation to determine $\Delta {\dot{V}}_{O_{2},ECMO}$ and $\Delta {\dot{V}}_{{\mathrm{CO}}_{2},ECMO}$ at the level of the membrane lung. No coefficients of variation in concentration levels are calculated over a period of time. Additionally, there is a short time delay between taking the pre-oxygenation and the post-oxygenation blood gas. Additionally, similarly to the traditional approach, the timing of the IC measurement at the natural lung is not totally in sync with the blood gas measurements. The second limitation is related to the BGA. In the model of Dash and Bassingthwaighte, 2,3-Bisphosphoglyceric acid is a necessary factor to perform the calculations, but this factor is not routinely measured in clinical practice [28]; therefore, normal values are chosen [27]. Finally, the same limitations, as described above, concerning traditional IC apply to MEEP as they use this technique to perform measurements at the level of the natural lung.

Another issue is that, in mechanically ventilated patients, the ventilation circuit needs to be disconnected to allow the connection with the indirect calorimeter. In particular, when the patient is infected with an airborne virus (e.g., COVID-19) this leads to major risk of infection for the staff. A possible solution is the usage of a plastic cover to limit virus spread; however, this does not eliminate the risk of infection completely [89].

Note that an ECMO patient mostly starts with the majority of gas exchange happening at the membrane lung, but as the patient progresses, the natural lung will gradually regain functionality. This will result in a shift of the amount of gas exchange at the membrane lung to more exchange at the natural lung. In other words, this is a dynamic system, hence it would be highly beneficial if the indirect calorimeter could measure the gas exchange at both sides continuously over the full dynamic range, namely an $F_{i,O_{2}}$ of 16–100%.

### 6.4. Limitations and Advantages of the Sensors Used in Indirect Calorimetry

The two types of oxygen sensors used in IC devices are paramagnetic oxygen sensors and GFCs, as described earlier. When comparing the two types both have their advantages and disadvantages. Firstly, according to literature, the response time of paramagnetic oxygen sensors is at least 1 order of magnitude faster than the response time of the GFC type [36,37,38,41,42,43], which could result in faster IC measurement times. However, manufacturers of commercial indirect calorimeters also claim high response times for the GFC-type sensor [52]. In addition, there is a further advantage of the paramagnetic variant over the GFC in terms of recalibration and lifetime. Since the modern version of the paramagnetic oxygen sensor almost does not degrade, no recalibration is necessary after factory calibration [90]. The major disadvantage of the paramagnetic oxygen sensor is its susceptibility to positional changes and external vibrations which can significantly impact the accuracy [91]. Another drawback is its cross-sensitivity to water vapor. Water vapor is diamagnetic, consequently it opposes the paramagnetic effect of $O_{2}$. A possible solution when using the paramagnetic oxygen sensor is to calibrate the sensor with an equally humid gas as the sample gas or to dry the gases before they enter the sensor [35]. The former is impractical but the latter solution could be achieved, as implemented in indirect calorimeters as the Deltatrac (Datex) and the Quark RMR (Cosmed), by using Nafion tubing which is permeable to water vapor [92]. Nafion tubing relies on the principle of differences in vapor pressure between the inside and the outside of the tube, i.e., when the vapor pressure on the inside is higher than the vapor pressure on the outside of the tubing, the moisture will diffuse out of the tubing and vice versa [93]. Contrarily, the GFC-type oxygen sensor is not cross-sensitive to water vapor [40] and also offers the advantage that it utilizes chemical reactions to produce its signal, eliminating the need for an external power source [94]. As opposed to the paramagnetic variant, the GFC needs to be recalibrated at periodic time intervals, typically 1 month, to ensure that the sensor is still working correctly [95]. This is due to the fact that the anode will oxidize over time. Exposure to higher levels of $O_{2}$ leads to faster degradation of the anode and, consequently, the analyzer will demand faster recalibration or reach complete failure quicker. Note that in ECMO-circuits the sweep gas can reach very high levels of $O_{2}$, up to 100%, consequently when using the traditional IC technique for patients on ECMO this would cause the GFC to degrade faster. Even when using MEEP, this phenomena may play a role as studies report that the $F_{i,O_{2}}$ settings of the mechanical ventilator are initiated at 100% and are reduced to <40% only when the partial pressure of $O_{2}$ in the blood stabilizes (after successive BGAs) [96]. The oxidation of the anode is the reason that the lifespan of the GFC is limited to typically 24 months in IC devices. In terms of accuracy, the datasheets of both types currently on the market report quiet similar values, 0.1 to <0.05% [46,50,51,97].

Regarding carbon dioxide analyzers, NDIR ${\mathrm{CO}}_{2}$ analyzers are the current sensors of choice to be used in indirect calorimeters. The first advantage is the extremely long lifetime of over 15 years due to the non-mechanical nature of the analyzer [98]. Secondly, the fast response time (dependent on flow rate) and the high amount of readings per second, with values reported up to 50 Hz, provide them with the ability to perform breath-by-breath measurements [99]. In the typical range of an exhaled breath (4–5%) NDIR ${\mathrm{CO}}_{2}$ sensors exist with an accuracy of ±2.9%, which is ±145 ppm if the exhaled breath contained 50,000 ppm ${\mathrm{CO}}_{2}$ [100]. Another strength lies in the extremely low power consumption, e.g., 3.5 mW at 3.3 V in the ExplorIR-M NDIR ${\mathrm{CO}}_{2}$ Sensor by SST Sensing [101]. This makes them very applicable to incorporate into portable devices. Limitations occur during general anaesthesia when nitrous oxide ($N_{2}O$) is used. $N_{2}O$ has an absorption band at 4.5 μm which is very close to the 4.3 μm of ${\mathrm{CO}}_{2}$; therefore, the analyzer is at risk of experiencing crosstalk leading to less accurate measurements [102].

The majority of the flow sensors used in indirect calorimeters consist of Pneumotachometers and turbine flow meters. An advantage of the Pneumotachometer over the turbine flow meter is that it has no moving mechanical parts, resulting in more robustness. The major limitation of the Pneumotachometer is that it is sensitive to changes in gas composition and temperature. This is due to the fact that viscosity, present in the formula of Hagen–Poiseuille (Equation (19)), is dependent on these parameters. A study by Turney et al. showed that the viscosity of room air (21% $O_{2}$) can be predicted by using Equation (20) and the viscosity of 99.6% $O_{2}$ by Equation (21) [103]. These equations originate from linear regression of known values of dynamic viscosities in literature over a temperature range of 20–40 °C [103,104].

(20) $$\mu_{room\;air}=174.4+0.4207\;\cdot \;T$$

(21) $$\mu_{99.6\%\;O_{2}}=199.9+0.2991\;\cdot \;T$$

*T* is the temperature in °C and the dynamic viscosity, *µ*, is expressed in microgram per centimeter per second. Due to the dependency on viscosity, the Pneumotachometer requires calibration each time $F_{i,O_{2}}$ changes to obtain the most accurate result [105,106]. The standard calibration technique is the iterative syringe stroke technique [107]. This calibration method uses the property that when a syringe with a specific volume, e.g., a volume of 3 L is commonly used, is emptied at variable speeds, the time-integration of the volumetric flow rate should equal the emptied volume of the syringe [106,108,109]. As IC measurements with commercial indirect calorimeters are performed during stable conditions, i.e., the system must deliver a stable $F_{i,O_{2}}$ [14,110], this issue is bypassed, but limits the use of IC. Additionally, humidity affects the Pneumotachometer because, as mentioned earlier, condensation increases the resistance of the resistive element. A significant benefit of the turbine flow meter over the Pneumotachometer is that the former has been reported to be almost insensitive to changes in gas composition from room air to 100% $O_{2}$, temperature changes and water vapor [111]. Regarding disadvantages of the turbine flow meter, Yeh et al. showed that turbine flow meters experience the “lag-before-start” and the “spin-after-stop” effect [111]. The “lag-before-start” effect means that at the beginning of inspiration or expiration the measurement signal of the turbine flow meter lags behind the actual signal and the “spin-after-stop” effect means that the rotor blades keep spinning at the end of expiration or inhalation. Both effects originate from friction and inertia-related matters and become worse at low flow rates [111,112]. These disadvantages render the turbine flow meter unreliable for breath-by-breath measurements as it fails in accurately detecting rapid changes in flow rate. Finally, non-disposable turbine flow meters are difficult to disinfect because the mechanical parts can be damaged resulting in loss of accuracy [60], but both types are available in disposable form making it easy to use in a clinical environment where the risk of contamination needs to be reduced [113].

## 7. Conclusions

In this paper, the theoretical aspects for measuring REE by means of IC were described. Subsequently, an overview has been given of the different techniques used/proposed for spontaneously breathing, mechanically ventilated, and extracorporeally oxygenated patients. The reader has also been provided with an overview of the different sensors used in these measurement systems. By understanding these theoretical aspects and comparing the various techniques and sensors, the advantages and limitations become clear in the context of IC.

IC is the gold standard for measuring REE leading to lower hospital mortality of critically ill-patients and better patient outcomes as literature suggests [5,114]. Despite the favorable clinical outcomes of IC, further advancements in technology could potentially still enhance the technique as it is yet impeded by notable limitations, particularly in the case of patients requiring high levels of oxygen supplementation exceeding 70%, such as possibly the case in those on mechanical ventilation and often the case in those on ECMO. The Haldane transformation employed by IC introduces errors in the measurement of REE in such patients, rendering the method unviable. Secondly, the presence of condensation in the sampling lines makes long term continuous IC measurements infeasible at the current time. In the specific case of ECMO patients, IC is not yet employed in routine clinical practice. Two techniques are currently proposed, but they still exhibit unresolved issues. The main problem with the traditional IC approach is that it lacks the synchronization of IC measurements taken at the natural and the membrane lung to determine $\Delta {\dot{V}}_{O_{2},tot}$ and $\Delta {\dot{V}}_{{\mathrm{CO}}_{2},tot}$. MEEP, on the other hand, is mainly limited by its reliance on two point measurements (pre- and post-oxygenation) taken at different times, which fail to provide a reliable measurement representative for a more general REE. In conclusion, a sensor network utilizing the traditional IC approach, capable of measuring over the full dynamic range of 16–100% $O_{2}$ in critically ill patients and that takes into account the time synchronization issue in ECMO patients, would be highly advantageous.

## Figures and Tables

**Table 1:** Overview of the technical specifications (measurement range, T90 response time, accuracy, and resolution) of paramagnetic and galvanic fuel cell oxygen sensors of commercial indirect calorimeters as reported by the manufacturers.

Name	Manufacturer	Type	Range	Response Time (T90)	Accuracy		Resolution	Reference
			%	ms	%vol	%	%vol	
Quark RMR	Cosmed	Paramagnetic	0–100	120	±0.05	±0.3	N.R. *	[47]
TrueOne2400	Parvo Medics	Paramagnetic	0–100	200	N.R.	±0.1	N.R.	[48]
Max-II and Max-IIa	AEI Technologies	Paramagnetic	0–100	N.R.	±0.03	N.R.	±0.01	[49]
Q-NRG and Q-NRG+	Cosmed	Galvanic Fuel Cell	0–75	N.R.	<±0.05	±0.3	±0.01	[50,51]
Ultima CPX	MGC Diagnostics	Galvanic Fuel Cell	0–100	<180	N.R.	±1	N.R.	[52]

* N.R. indicates not reported.

**Table 2:** Overview of the technical specifications (measurement range, T90 response time, accuracy and resolution) of NDIR carbon dioxide sensors of commercial indirect calorimeters, as reported by the manufacturers.

Name	Manufacturer	Range	Response Time (T90)	Accuracy		Resolution	Reference
		%	ms	%vol	%	%vol	
Quark RMR	Cosmed	0–10	100	±0.05	±1	N.R. *	[47]
TrueOne2400	Parvo Medics	0–15	100	N.R.	±0.1 (for 0–10% ${\mathrm{CO}}_{2}$)	N.R.	[48]
Max-II and Max-IIa	AEI Technologies	0–10	N.R.	±0.03	N.R.	±0.01	[49]
Q-NRG and Q-NRG+	Cosmed	0–10	N.R.	±0.05	±1	±0.01	[50,51]
Ultima CPX	MGC Diagnostics	0–15	<180	N.R.	±1 (for 0–10% ${\mathrm{CO}}_{2}$)	N.R.	[52]

* N.R. indicates not reported.

**Table 3:** Overview of the technical specifications (measurement range and accuracy) of turbine flow meters and Pneumotachometers of commercial indirect calorimeters as reported by the manufacturers.

Name	Manufacturer	Type	Range	Accuracy		Reference
			L/min	%	mL/s	
Quark RMR	Cosmed	Pneumotachometer	0–960	±2	±20	[47]
		Turbine	0–360	±2	±20	
TrueOne2400	Parvo Medics	Pneumotachometer	0–800	±2	N.R. *	[48]
Max-II and Max-IIa	AEI Technologies	Pneumotachometer	0–800	<±1	N.R.	[49]
Q-NRG	Cosmed	Turbine	0–360	±2	±20	[50]
Q-NRG+	Cosmed	Pneumotachometer	0–960	±2	±20	[51]
		Turbine	0–360	±2	±20	

* N.R. indicates not reported.

## Data Availability

The data presented in this study are available on request from the corresponding author.

## Abbreviations

The following abbreviations are used in this manuscript:

ASPEN	American Society for Parental and Enteral Nutrition and Metabolism
BGA	Blood gas analysis
${\mathrm{CO}}_{2}$	Carbon dioxide
ECMO	Extracorporeal Membrane Oxygenation
ESPEN	European Society for Clinical Nutrition and Metabolism
GFC	Galvanic fuel cell
IC	Indirect calorimetry
MEEP	Measuring Energy Expenditure in extracorporeal lung support Patients
$N_{2}$	Nitrogen
NDIR	Non-dispersive infrared
$O_{2}$	Oxygen
REE	Resting energy expenditure
RQ	Respiratory quotient
SCCM	Society of Critical Care Medicine
UN	Urinary nitrogen

## Symbols

The following symbols are used in this manuscript:

c	Concentration (mol · m${}^{-3}$)
D	Diameter (m)
F	Volume fraction (%*v*/*v*)
I	Light intensity (W · m${}^{-2}$)
L	Length (m)
m	Mass (kg)
$\dot{m}$	Mass flow rate (kg · s${}^{-1}$)
p	Fraction of caloric production due to protein metabolism (%)
P	Pressure (Pa)
t	Time (s)
T	Period (s)
T	Temperature (°C)
T	Transmittance (-)
$\dot{V}$	Volumetric flow rate (m${}^{3}\;\cdot \;$ s${}^{-1}$)
ε	Relative error (%)
ϵ	Molar absorption coefficient (m${}^{2}\;\cdot \;$ mol${}^{-1}$)
µ	Dynamic viscosity (Pa · s)

## Appendix A. Derivation of the Weir Formula

The total amount of $O_{2}$ consumed, $\Delta V_{O_{2}}$, is given by the sum of partial amounts of $O_{2}$ used for metabolizing carbohydrate $V_{O_{2},carb}$, protein $V_{O_{2},prot}$, and fat $V_{O_{2},fat}$, resulting in Equation (A1).

(A1) $$\Delta V_{O_{2}}=V_{O_{2},carb}+V_{O_{2},prot}+V_{O_{2},fat}$$

The total amount of ${\mathrm{CO}}_{2}$ produced, $\Delta V_{{\mathrm{CO}}_{2}}$, can be determined by using the values for the RQ, which equals the ratio of ${\mathrm{CO}}_{2}$ produced and $O_{2}$ consumed, as depicted in Table A1.

The corresponding amount of produced ${\mathrm{CO}}_{2}$ is calculated resulting in Equation (A2).

(A2) $$\Delta V_{CO_{2}}=V_{O_{2},carb}+0.802V_{O_{2},prot}+0.718V_{O_{2},fat}$$

By using the values for energy per volume $O_{2}$ metabolized, also depicted in Table A1, the REE can be calculated, as shown in Equation (A3).

(A3) $$REE=5.047V_{O_{2},carb}+4.463V_{O_{2},prot}+4.735V_{O_{2},fat}$$

By substituting Equation (A1) in Equations (A2) and (A3) the following expressions are obtained:

(A4) $$\Delta V_{CO_{2}}=\Delta V_{O_{2}}-0.198V_{prot}-0.282V_{fat}$$

(A5) $$REE=5.047\Delta V_{O_{2}}-0.584V_{prot}-0.312V_{fat}$$

Subsequently, Equation (A4) is substituted in Equation (A5) resulting in Equation (A6) in which the term $-0.365\;V_{prot}$ can be regarded as the correction factor for protein metabolism [12].

(A6) $$REE=3.941\Delta V_{O_{2}}+1.106\Delta V_{CO_{2}}-0.365V_{prot}$$

The first method for quantifying the protein correction factor is based on the fact that 5.941 L of $O_{2}$ needs to be consumed for the production of 1 g of UN resulting in Equation (A7) [11].

(A7) $$REE=3.941\Delta V_{O_{2}}+1.106\Delta V_{CO_{2}}-2.17m_{UN}$$

The second method for determining the protein correction factor requires a new parameter p, which represents the fraction of caloric production due to protein. Together with the data from Table A1, the volume of $O_{2}$ consumed for protein metabolism can be calculated as shown in Equation (A8).

(A8) $$V_{prot}=\frac{p\;\cdot \;REE}{4.463}$$

This results in Formula (A9) for determining REE.

(A9) $$REE=\frac{3.941\Delta V_{O_{2}}+1.106\Delta V_{CO_{2}}}{1-0.0818p}$$

**Table A1:** Numerical values of RQ and energy per volume $O_{2}$ metabolized for carbohydrates, proteins, and fats adapted from [12].

	Carbohydrate	Protein	Fat
RQ	1	0.802	0.718
Energy per volume $O_{2}$ metabolized (kcal/L)	5.047	4.463	4.735

## Appendix B. Derivation of the Haldane Transformation

The Haldane transformation relies on the assumption that $N_{2}$ is inert and does not take part in the eventual gas exchange. Mathematically, this translates to Equation (A10).

(A10) $${\dot{V}}_{i}\;\cdot \;F_{i,N_{2}}={\dot{V}}_{e}\;\cdot \;F_{e,N_{2}}$$

The next assumption that is made is that the exchanged gases consist of only $O_{2}$, ${\mathrm{CO}}_{2}$, and $N_{2}$ so that Equations (A11) and (A12) can be used.

(A11) $$F_{e,N_{2}}=1-F_{e,O_{2}}-F_{e,CO_{2}}$$

(A12) $$F_{i,N_{2}}=1-F_{i,O_{2}}-F_{i,CO_{2}}$$

By substitution of Equations (A11) and (A12) in Formula (A10), a useful expression to calculate ${\dot{V}}_{i}$ is derived as shown in Equation (A13).

(A13) $${\dot{V}}_{i}={\dot{V}}_{e}\left(\frac{1-F_{e,O_{2}}-F_{e,CO_{2}}}{1-F_{i,O_{2}}-F_{i,CO_{2}}}\right)$$

Finally, by further substituting Equation (A13) in Equations (4) and (5) the final expressions to calculate $\Delta {\dot{V}}_{O_{2}}$ and $\Delta {\dot{V}}_{{\mathrm{CO}}_{2}}$ are derived as shown in Equations (A14) and (A15), respectively.

(A14) $$\Delta {\dot{V}}_{O_{2}}={\dot{V}}_{e}\left[\left(\frac{1-F_{e,O_{2}}-F_{e,CO_{2}}}{1-F_{i,O_{2}}-F_{i,CO_{2}}}\right)F_{i,O_{2}}-F_{e,O_{2}}\right]$$

(A15) $$\Delta {\dot{V}}_{CO_{2}}={\dot{V}}_{e}\left[F_{e,CO_{2}}-\left(\frac{1-F_{e,O_{2}}-F_{e,CO_{2}}}{1-F_{i,O_{2}}-F_{i,CO_{2}}}\right)F_{i,CO_{2}}\right]$$
